# State of Lunacy in Scotland
*"Report of her Majesty's Commissioners," pp. 257; with an Appendix, pp. 581. Presented to both Houses of Parliament. Edinburgh: T. Constable. 1857.


**Published:** 1857-07-01

**Authors:** 


### Art. III.—STATE OF LUNACY IN SCOTLAND.*

These official volumes—the last-named being rather voluminous—are the result of a Royal Commission, dated 3rd April, 1855, which was issued by her Majesty to inquire into the condition of public asylums in Scotland, as well as of the existing state of the law of that country in reference to lunatics, and the institutions for the insane. Various important subjects are discussed by the gentlemen who were appointed to undertake the onerous duties assigned to them; and when it is mentioned that the Commissioners were Mr. Monteith, Sheriff-Depute of Fifeshire; Dr. James Coxe, a Fellow of the College of Physicians in Edinburgh; with Mr. Gaskell and Mr. Campbell, two of the Commissioners in Lunacy for England,—such selection is a sufficient guarantee the searching investigations thus instituted would prove both instructive and of the highest moment. In drawing up the minute Report now lying before us, the Royal Commissioners divide their inquiry into six separate heads, which comprise—

"1. An abstract of the existing law of Scotland on the subject of lunacy, both as regards the custody and treatment of the persons of lunatics, and the care and management of their property.—2. A statement of the numbers of lunatics at present in Scotland, and of the manner in which they are distributed.—3. A description of the nature and extent of the accommodation provided for the insane, whether in public asylums, or private establishments recognised by law; together with an account of the condition of these establishments, and of the treatment of the lunatics confined in them.—4. An account of the condition of lunatics not confined in any of these establishments, in so far as we have been able to ascertain the same.—5. An exposition of the mode in which the law has been, and is practically administered, having special reference to the question, how far any abuses that may be found to exist, are owing to the defective administration of the present law, or may require new legislative enactments for their effectual remedy.—Lastly, A brief resumption of the leading particulars which seem to call for legislative interference; and of the principles on which it appears to us that such remedial legislation ought to be based."

These varied matters are all elaborately discussed; and believing many points must greatly interest not only professional readers, but philanthropists generally, we propose, even at the risk of becoming tedious, to make an analysis explaining the principal questions mooted in the above-named Report. Prior, however, to undertaking this rather laborious task, it seems but simple justice to one of our own respected contributors to ob-

* "Report of her Majesty's Commissioners," pp. 257; with an Appendix, pp. 581. Presented to both Houses of Parliament. Edinburgh: T. Constable. 1857.

serve cursorily that, in three numbers of the "Psychological Journal" for 1856, much similar matter respecting the public lunatic asylums and the lunacy laws of Scotland have already appeared from the pen of Dr. Webster, who had also visited the public insane institutions now reported upon by her Majesty's Commissioners, and, what is worth mentioning, during the same season; whose Notes contained not only a concise account of the chief legal regulations affecting lunatics in North Britain, but likewise described the then existing condition of the identical Scottish establishments for mad patients which are elaborately noticed in the Parliamentary document now published. To use the words of a Scottish medical jurist, when alluding to these judicial observations, we can justly say, the summary in our Journal then given of the law and practice in reference to lunatics, although brief, is admirable, very correct, and complete. Indeed, there is no work on medical jurisprudence now in the hands of the profession, where the legal information thus given can be obtained; hence it becomes of great value, and constitutes a most able production; while an analogous combination of extent, with brevity and accuracy of information, appears too rarely available. Having made the above general remarks regarding the labours of our former correspondent, in again reverting to the volumes whose title heads this article, we would just observe, with reference to the law of Scotland affecting lunatics, that thinking it would seem almost supererogation for us again to go over the subject thus so recently brought under the notice of our readers, we therefore at once pass on to another division of the Report—viz., the number and distribution of the insane.

According to official returns, the total amount of insane persons ascertained as now existing throughout Scotland appears to be 7403, and are thus classified—viz.:

| | |
|---|---|
| "Under special protection of the law . . . | 3736 |
| In poorhouses, but not under Sheriff's warrant . | 253 |
| With relations or strangers, or living alone . | 3798 |
| In unlicensed establishments . . . . | 24 |
| Total . . . . | 7403 |

"We now proceed to show the proportion of the males to the females, the proportion of lunatics to congenital cases of idiocy, the social position of the patients, and the probable proportions of the curable and incurable.

"It appears that of the 7403 patients, there are—

| | |
|---|---|
| Males . . . . . . . . . . | 3736 |
| Females . . . . . . . . . . | 3667 |
| Total . . . . | 7403 |

| | |
|---|---|
| Congenital idiots | 2603 |
| Lunatics | 4800 |
| Total | 7403 |
| Private patients | 2732 |
| Paupers | 4642 |
| Criminals | 29 |
| Total | 7403 |

Again, it is also most important to add, that—

"The total number of the insane in Scotland would thus be divided into:

| | |
|---|---|
| Curable lunatics | 768 |
| Incurable lunatics | 4032 |
| Congenital idiots and imbeciles | 2603 |
| Total | 7403 |

"The distinction thus drawn, however, can only be regarded as a vague approximation to the truth.

"In investigating the number and condition of the insane, certain facts were elicited to which it may be proper to advert under this head, as they have a bearing more or less direct on the main object of our inquiry.

"The first of these is the very large proportion of lunatics in the pauper population as compared with the other classes of the community.

"According to the returns of the Board of Supervision, the number of paupers in Scotland, registered on the poor-roll, on the 14th of May, 1855, amounted to 79,887, while the number of insane and fatuous poor, included in this number, was 3904. In other words, a population of 79,887 paupers yielded more than one-half of the whole number of the insane of the kingdom, showing the powerful affinity that exists between poverty and mental disease. Each is reciprocally productive of the other, and alternately cause and effect. A person of feeble or diseased brain, if left to his own resources, naturally sinks in the social scale, and is ultimately reduced to a state of pauperism. On the other hand, the cares that attend poverty, in conjunction with the deteriorating agency of scanty and innutritious food, have a powerful influence in weakening the mental powers, and inducing insanity. The degree in which these two causes operate is, however, widely different; and there is reason to think, that more individuals are reduced to poverty through a defective mental constitution, than are rendered insane through poverty.

"Another fact, painfully illustrative of the evils resulting from the imperfect provision for pauper lunatics, is the number of idiotic women who have borne illegitimate children, and whose mental defect is frequently manifested in their offspring. These we have ascertained to amount to not less than 126; and there is cause to believe, that many cases of this description have escaped observation; and, also, that the fact of such weak-minded females having given birth to offspring, has often, for obvious reasons, been designedly concealed. Accordingly, large as the above number appears, we are satisfied it should be taken considerably higher. It must be kept in view, too, that many of these women have given birth to several children."

Another interesting feature equally deserves special mention—namely,

"The proportion which the number of lunatics bears to the general population, as well as the modification of their proportions according to differences of locality and other circumstances, fall naturally to be considered under this head of our Report. The total population of Scotland, by the census of 1851, was 2,888,742, while the insane of every class amounted, on the 14th May, 1855, to 7403, yielding a proportion of 2.562 per 1000, or one insane person to every 390 of the population. This, however, can only be regarded as a probable approximation, owing to the necessity we were under of taking the population as in 1851 for the basis of our calculations, while the number of the insane returned is as in May, 1855.

"Here a very interesting question presents itself—namely, whether the ratio of lunatics to the general population is increasing or diminishing. There is an idea very generally prevalent, that the number of the insane, in proportion to the rest of the community, is decidedly on the increase, and that this result is chiefly attributable to over-exertion of the mental faculties, consequent on the cares and struggles attendant on civilization. The principal reason for this opinion is, the always increasing pressure for the admission of patients into asylums. But the question here occurs,—Does this pressure really arise from an increase in the numbers of the insane, or is it caused by more attention being directed to their care and treatment? We have no previous enumeration of the insane in Scotland, and have, therefore, no standard by which to determine, whether their number has increased in a greater ratio than that of the rest of the community; but, even if this were found to be the case, we should not be authorized in ascribing the result to the increased activity and greater strain of the mental powers that accompany civilization. The question cannot be decided by an appeal to statistical returns, for we have not information sufficient to enable us to institute any useful comparison between the proportional numbers of the insane in those counties which have most advanced in civilization, and in those which have lagged behind.

"For, in the first place, in large cities, where there is the greatest mental activity, there, also, is the greatest physical deterioration. The energies of the working population are wasted by continuous labour, while their physical condition is lowered by a residence in unwholesome dwellings, and by the abuse of stimulating liquors to restore their exhausted powers. Experience shows that these combined influences constitute a prolific source of insanity among the crowded population of our towns. And, secondly, in those counties where thought most stagnates, a large proportion of the cases of mental disease is due to congenital causes. The population, unaffected by extraneous influences, intermarry among themselves, and the hereditary taint which is thus engendered shows itself unmistakeably in the large proportion of idiots and imbeciles. The preponderance of this cause of mental disease in remote counties distinctly appears, on comparing the proportions of congenital cases occurring in them with those found in southern counties, where the mental powers have been more called into action, and intermarriage is less frequent. This is shown in the following tables:—

TABLE I.

*Counties remote from Influences that incite to Mental Activity.*

| Counties. | Population. | Number of Cases of Congenital Mental Disease. | Proportion of Congenital Cases per 10,000 of Population. |
|---|---|---|---|
| Argyle . . . . . | 89,298 | 133 | 14.8939 |
| Caithness . . . . | 38,709 | 92 | 23.7670 |
| Inverness . . . . | 96,500 | 91 | 9.4300 |
| Orkney and Shetland. | 62,533 | 177 | 28.3050 |
| Ross and Cromarty . | 82,707 | 102 | 12.3326 |
| Sutherland . . . . | 25,793 | 24 | 9.3048 |
| Average. . . . | 65,923 | 103 | 15.6242 |

TABLE II.

*Counties exposed to Influences that incite to Mental Activity.*

| Counties. | Population. | Number of Cases of Congenital Mental Disease, | Proportion of Congenital Cases per 10,000 of Population. |
|---|---|---|---|
| Dumbarton . . . . | 45,103 | 34 | 7.5383 |
| Edinburgh . . . . | 259,435 | *160 | 6.1672 |
| Forfar . . . . . | 191,264 | *121 | 6.3263 |
| Lanark . . . . . | 530,169 | *129 | 2.4331 |
| Renfrew . . . . . | 161,091 | *53 | 3.2901 |
| Stirling . . . . . | 86,237 | 35 | 4.0585 |
| Average. . . . | 212,216 | 89 | 4.1938 |

"It thus appears that a Highland population contains more than three times the number of congenital cases of mental disease found in an equal Lowland population; and that the difference becomes much greater, if the comparison be confined to single counties of the two series—as, for instance, to Lanarkshire, and Orkney and Shetland, as extreme representatives of the two classes of counties.

"While, therefore, our inquiries afford no means of deciding whether or not the number of insane is actually on the increase, they afford us grounds for thinking that civilization, which leads to an improved condition of the people, is not productive of insanity."

* All the cases of idiocy in the asylums and poorhouses of those counties ar included in the above numbers, though many of them are from other counties.

With reference to a very important question, exhibiting the distribution of the insane, the various kinds of domiciles appropriated for their reception may next be mentioned. By the returns obtained from official parties, it appears—

"I. The houses in which the insane are received, under cognizance of the Sheriff, consist of—

1. Chartered asylums.
2. Public asylums without a charter.
3. Poorhouses receiving patients either in separate wards, or in common with ordinary paupers.
4. Private licensed houses.
5. Houses for single patients reported to the Sheriff.
6. Prisons.
7. Schools for idiots.

"On the 14th May, 1855,* the insane, under the special guardianship of the law, were distributed in the various houses as follows:—

| | |
|---|---|
| In chartered asylums . . . . . . | 2123 |
| „ public asylums without charter . . . | 40 |
| „ licensed poorhouses . . . . . . | 423 |
| „ licensed private houses . . . . . | 657 |
| „ reported houses . . . . . . . | 41 |
| „ prisons . . . . . . . . | 29 |
| „ schools for idiots . . . . . . . | 15 |
| Total . . . . | 3328 |

"II. The houses in which the insane are received without the cognizance of the Sheriff are—

1. Unlicensed poorhouses.
2. Private houses not reported to the Sheriff in terms of the law, and houses of relatives.
3. Unlicensed private establishments.

| | |
|---|---|
| The number of the insane in unlicensed poorhouses on 14th May, 1855, was . . . . . . | 253 |
| In private houses not reported to the Sheriff, and houses of relatives . . . . . . . | 3798 |
| In unlicensed private establishments . . . . | 24 |
| Total . . . . . | 4075 |

"The 2732 private patients were distributed as follows:—

| | |
|---|---|
| In chartered asylums . . . . . . . | 652 |
| „ licensed houses . . . . . . . | 231 |
| „ poorhouses . . . . . . . | 9 |
| „ reported houses . . . . . . . | 10 |
| „ schools for idiots . . . . . . . | 12 |
| „ unlicensed establishments . . . . . . | 18 |
| With relatives . . . . . . . . | 1453 |
| With strangers . . . . . . . . | 297 |
| Not under care of any one . . . . . . | 50 |
| Total . . . . . | 2732 |

* This date does not apply to the School for Idiots in Edinburgh, from which the returns were obtained at a later period.

"The 4642 paupers were disposed of as follows:—

| | |
|---|---|
| In chartered asylums . . . . . . . | 1511 |
| „ licensed houses . . . . . . . | 426 |
| „ poorhouses . . . . . . . . | 667 |
| „ reported houses . . . . . . . | 31 |
| „ schools for idiots . . . . . . . | 3 |
| „ unlicensed establishments . . . . | 6 |
| With relatives . . . . . . . . | 1217 |
| With strangers . . . . . . . . | 640 |
| Not under care of any one . . . . . . | 141 |
| Total . . . . . | 4642 |

"The constabulary returns show that there are 3607 single patients resident with relatives, or placed under the charge of strangers, and that they are distributed as follows—viz.

Patients not paupers—

| | |
|---|---|
| With relatives . . . . . . . . | 1453 |
| With strangers . . . . . . . . | 297 |
| Total . . . . . | 1750 |

Paupers—

| | |
|---|---|
| With relatives . . . . . . . . | 1217 |
| With strangers . . . . . . . . | 640 |
| Total . . . . . | 1857 |

"As many as 191 patients are reported to have no one in charge of them. Of these, 50 are independent of parochial relief, and 141 are paupers."

In regard to the accommodation supplied by public institutions, there are in Scotland, at present, seven chartered Asylums—namely, 1. The Royal Asylum, Aberdeen; 2. The Crichton Institution, Dumfries, including the Southern Counties Asylum; 3. The Royal Asylum, Dundee; 4. The Royal Asylum, Edinburgh; 5. The Royal Asylum, Glasgow; 6. The Royal Asylum, Montrose; and, 7. James Murray's Royal Asylum, Perth. Respecting each of which institutions we now proceed to transcribe, from the Commissioners' Report, some of the chief features characterizing these different public establishments, with various statistical details, in order to illustrate their actual working and recent condition. However, before specifically noticing the respective institutions, we would quote the following introductory remarks made by the Commissioners:—

"The existing accommodation provided for the insane in Scotland, is a proof of the interest there excited by this most destitute portion of the community. For, though no legislative enactments have compelled the erection of asylums for pauper lunatics in this portion of the kingdom, we find here several large institutions, founded, and in a great measure maintained, by the exertions and benevolence of private individuals. These institutions, commonly called chartered asylums, from the circumstance that each has a royal charter of incorporation, are designed not only for the reception of pauper patients, but also of those whose means enable them to defray the expense of care and treatment suitable to a higher station in life. They also afford some assistance to those reduced in circumstances, who, from their previous habits, can appreciate, but are not able to pay for, a better style of accommodation.

"There are, at the present time, seven establishments of this nature, capable of admitting 572 private, and 1522 pauper patients.

"Although this amount of accommodation does not, by any means, meet the requirements of the country, yet it is satisfactory to find that so large a provision has been voluntarily made; and we would fain hope that this fact indicates the existence of such an amount of solicitude for the insane, as will lead, ere long, to the establishment of sufficient accommodation to supply the wants of the country in every district.

"It is due to all parties concerned in establishing and conducting the chartered asylums in Scotland, to express our high admiration of the motives which have led them to provide such means of succour and restoration for the insane. We have reason to believe that no country, proportionately to its population, has voluntarily done so much for this class of sufferers; and, although it may be said that Scotland presents an unfavourable contrast with most civilized States, in not having any national institutions for the reception of its insane poor, yet, as regards those erected by private benevolence, it may claim a marked and honourable distinction.

"An important fact is deducible from the histories which we have thus collected, namely, that the building and grounds being provided, the institution becomes self-supporting, the payments made for the care and treatment of the patients covering all current expenses.

"In reference to their origin, it will be observed, that when hospitals for the insane have been connected with infirmaries, they have always sprung from the latter, as subsidiary establishments, except in the case of Montrose, which presents a remarkable instance to the contrary. Here the hospital for mental affections has been placed in a primary, and that for bodily complaints in a secondary position."

These observations are highly creditable to the country respecting whose proceedings they thus speak, and we now reproduce them thus prominently in our own pages, so as to excite the emulation of other but more wealthy kingdoms, where compulsory assessments are often the usual means employed in order to procure adequate accommodation and appropriate treatment for the pauper insane.

Respecting the site, size, and construction of the chartered asylums, the Commissioners make the following judicious criticisms, which well deserve careful perusal, both on account of being apposite to various questions now under discussion, and likewise as they convey a very good general idea of the different institutions described:—

"The important influence exercised upon the inmates of asylums, by the nature of the sites upon which they are built, can hardly be over-estimated. With the exception of the institution at Montrose, the sites of all the chartered asylums are well selected, although few possess sufficient land to afford full agricultural employment for the male patients.

"They are generally placed in elevated situations, commanding agreeable prospects, and, although at a sufficient distance from a town to secure privacy to the inmates, and to enable the patients to take undisturbed exercise beyond the limits of the premises, yet they are not so remote as to shut out the officials and servants from general society, nor to prevent the more trustworthy patients from enjoying the benefit of an occasional visit to the public amusements of a city.

"The size of an asylum has a considerable influence upon the condition of the patients; and it may be considered as a settled rule, that, everything else being equal, moderate-sized asylums can be more efficiently conducted than large institutions.

"It will be seen by reference to Appendix B, that the chartered asylums vary considerably in size,—the largest, that of Edinburgh, containing accommodation for 467 patients, whilst the Perth asylum only accommodates 183. In addition to the advantages to be derived from treatment in an asylum of moderate size, there can be no doubt that large central establishments are not so well adapted to meet the wants of the community as smaller local asylums, to which patients could be readily sent. Besides, we have reason to believe that the patients themselves prefer the smaller houses, where their individuality is more recognised, and where they have a more home-like feeling.

"Most of the chartered asylums are well constructed, and afford good accommodation to the inmates. In some, however, faulty arrangements are observable, such as double galleries, stone floors, deficient means of warming and ventilating, and objectionable arrangements for the seclusion of refractory patients in dark rooms. In a few of the houses, two existing peculiarities are worthy of remark,—namely, central inspection staircases, and open spaces on the upper stories,

"The Asylum of Perth, and the Crichton Institution at Dumfries, are constructed upon a somewhat similar plan. Each has a central staircase, with a curiously contrived double wall; and the galleries, which radiate from the staircase, can all be inspected through glazed apertures over the doors."

The constitution and management of the public insane establishments of Scotland next come under observation. According to the Report—

"The management of the chartered asylums is conducted by Boards of Directors appointed under the charters of incorporation. They consist of directors ex-officiis, life directors, and, frequently, also of annual directors; the appointment of the last taking place at the annual general meeting of the contributors. As a general rule, the directors ex-officiis take little part in the business of the asylum, and, as the annual meetings of contributors are generally very thinly attended, the management has a tendency to lapse into the hands of a few individuals. The directors, so constituted, are thus, without any blame being imputable to them, in a great degree, an irresponsible body, there being little or no check on their proceedings, on the part of their constituents or the public. Generally, the management has been ably conducted; but, occasionally, mistakes have been committed, through which the patients have not enjoyed all the benefits which otherwise might have been afforded them. In the case of the Glasgow Asylum, for instance, the directors, in their zeal to extend the benefits of the institution, have incurred a debt of nearly 40,000*l.*, which presses heavily upon its resources, and impedes improvements which the medical superintendent is desirous to introduce. From the statements made by the secretary of the Edinburgh Asylum, there is sôme reason to fear that the directors of that institution are about to fall into a similar error. Although we think it necessary to allude to the pecuniary embarrassments of these asylums, there can be no doubt that the position of their directors has been one of peculiar difficulty; for they were left to decide between extending the accommodation beyond the limits warranted by the state of their finances, or resisting the continually increasing demands for admission. In deciding on the former course, therefore, they were actuated solely by motives of humanity, and were, at the same time, almost certain of incurring great personal trouble and responsibility."

With regard to medical surveillance, and power which official professional gentlemen possess, the Commissioners observe—

"The medical superintendence of the chartered asylums is, as a rule, confided to a resident physician, who is appointed by the directors, and is removable by them. His powers, in regulating the treatment of the patients, are virtually plenary, though subject to the control of the visiting committee of the directors, and occasionally, as at Perth and Dundee, to that of a non-resident, or consulting physician."

Although the resident medical superintendent in most instances has considerable directing power, he should nevertheless have more administrative influence than he often actually possesses; indeed, he ought to exercise *paramount* authority in everything appertaining to the management, and moral, medical, or physical treatment of patients under his charge. In fact, he must be like the colonel of a regiment. He should further be invited to attend all meetings of managing committees, although without the privilege of voting—being a salaried officer—in order that he might thus give opinions respecting the admission of new patients, or upon any professional questions which then arose regarding matters connected with the institution or its inmates, as also to prevent future misunderstandings. This system is advantageously pursued at Bethlem Hospital, where, during all weekly meetings held for the reception of patients, their discharge, and so forth, the resident physician constantly attends, and sits with the committee. The same mode of proceeding ought to be followed at all public insane asylums, since it could not but facilitate business, and be otherwise beneficial; while the medical superintendent would thereby occupy, virtually before the world, the position to which he is entitled, as well from professional rank and scientific attainments, as on account of the responsible office he actually fills in such establishments.

Respecting the all-important question of instrumental coercion and seclusion, the Report contains the following pertinent remarks:—

"Personal restraint, by the application of the strait-waistcoat, or of straps or muffs, is almost entirely banished from the chartered asylums, but we have reason to think that seclusion for long periods is frequently used. This remark applies more especially to the asylums of Montrose, Glasgow, Aberdeen, and Edinburgh. The necessity for the use of lengthened seclusion is mostly due to faults of construction in the house, to overcrowding, to deficiency in the means of exercise, and to the want of a sufficient number of attendants; causes which are, to some extent, beyond the power of the medical superintendents to remove."

"The statutes not requiring any record to be made of the confinement to which patients may be subjected in seclusion-rooms, we are unable to report with accuracy as to the prevalence of the practice, or to make any comparative estimate of the extent to which this species of restraint has been resorted to. It is to be regretted that such written accounts are not generally kept; for there can be little doubt that if an entry of each instance of seclusion, and also of the reason assigned for resorting to it, had been made necessary by law, a powerful means of checking and diminishing its use would have been established. The requirements of the Legislature having been satisfactorily observed in the chartered asylums, this would, in all probability, have been the result of such a register of seclusion in these institutions; but as respects licensed houses, where an utter disregard has been shown of the clear, precise, and stringent regulations already enacted as to the use of mechanical restraint, little benefit would have arisen from any further enactment for recording this mode of restricting the liberty of the patients."

Occupation of patients, and the modes usually adopted for their amusement, are also noticed. Regarding the latter, it is stated that

"In several of the asylums, very much has been done to afford recreation and amusement to the patients. Various sports and games have been introduced, and in most of the houses there are frequent excursions, and occasional pic-nics, concerts, lectures, evening parties, and dances. In that of Dumfries, there are also theatrical performances.

"The asylum in which least has been accomplished in this respect, is that of Aberdeen.

"While fully recognising the importance of recreation and amusement, we are disposed to think that the efforts of some medical superintendents have been extended too much in this direction, to the exclusion of more serious occupations. Simple amusement can never dispel *ennui*, nor afford the same amount of healthy occupation to the mind as useful and productive labour.

"In most of the chartered asylums, there is a want of objects of every-day interest, calculated to afford quiet pleasure and enjoyment, which might be supplied at very little cost. The providing of such objects is not a matter of indifference, for they tend to draw the patient's mind from its morbid thoughts, and to prepare the way for recovery.

"At Dumfries, in this respect, as well as in every other that tends to alleviate the condition of the patients, a great deal has been accomplished. This asylum contains a museum of specimens in natural history, and also a library consisting of about 5000 volumes. Here, and also at Morningside, periodical publications are regularly printed and circulated, many of the articles being contributed by the patients themselves."

The Commissioners subsequently discuss other interesting points connected with the management and actual results obtained at different public asylums, but into which our limited space prevents us now entering. We, therefore, can only transcribe the following summary of their observations:—

"Reviewing generally the condition of the chartered asylums, it is gratifying to be able to report that they are in many respects in a highly satisfactory state, and that the large amount of accommodation which they afford to private patients is duly appreciated by the public.

"It appears that of the 833 private patients placed in asylums in Scotland, 652 are in chartered asylums, and only 231 in licensed houses.

"The contrast in this respect, between Scotland and the southern portion of the United Kingdom, is worthy of observation.

"On reference to the last Report of the Commissioners in Lunacy, it appears that of the 4442 private patients in asylums in England and Wales, as many as 2746 are in private asylums, and 1696 are in public hospitals; this latter number comprising 669 patients in Bethlem, St. Luke's and Guy's Hospitals, and the Institution for Idiots. After deducting these, therefore, it appears that in English hospitals, which are analogous to the chartered asylums in Scotland, the private patients amount to only about 1000, whereas as many as 2746 are in licensed houses. That this state of matters arises, at least in some degree, from the want of a larger amount of this kind of accommodation in England, may be inferred from the fact that a large proportion of the private patients in the Crichton Institution are natives of England.

"The gentlemen who undertake the responsible duties of governors or directors in the chartered asylums, devote much time and consideration to the general management of their respective establishments; and they manifest an earnest desire to promote the welfare and consider the comfort of the inmates, and to advance the interests of the institutions over which they preside.

"The treatment of the patients is liberal and judicious; and, notwithstanding existing difficulties and obstacles to improvement, their condition is, on the whole, deserving of commendation.

"The treatment adopted towards the educated classes is, in many respects, very praiseworthy. In addition to the means employed to diversify the daily course of life, and to break through the monotony and routine too common in most lunatic asylums, the patients have the benefit of frequent, and, occasionally, of extended excursions; and, in a few instances, houses have been taken at the sea-side for the use of the patients during the summer months.

"All the chartered asylums have comprehensive codes of printed rules and regulations. Annual accounts of receipts and expenditure, as well as reports of the management and treatment of the patients, are published, giving full details of the proceedings, in each establishment, during the year.

"Various records, not required by statute, are generally kept, among which is a casualty-book, and also a case-book, containing a statement relative to each patient, showing the origin, course, and duration of the disease previous to admission, and the subsequent treatment adopted in the asylum. Considerable care is also taken to ascertain full particulars respecting the previous condition of the patients; and, for this purpose, printed questions, designed to elicit information, are issued to applicants for the admission of patients.

"The chartered asylums of Scotland are superintended by experienced medical gentlemen, of high standing in their profession, aided in the larger establishments by able assistants. From their size, and capabilities of receiving a considerable number of better-class patients, they are able to command the services of accomplished practitioners; and, in this respect, they have a manifest advantage. In the generality of them, nevertheless, an obvious evil results from the congregation of patients belonging to various grades of society in the same institution. A minute separation of the inmates into classes, both as respects position in life, as well as the nature of the malady, becomes necessary; and, consequently, the patients are subdivided into a large number of communities, each having their respective apartments and airing-grounds. By the adoption of such arrangements, liberty within doors is diminished, the facilities of egress into the open air are impeded, and the space appropriated for exercise is considerably curtailed; and the general results are isolation of individuals belonging to the upper classes, restricted exercise to the inmates generally, and lengthened seclusion of the more refractory patients.

"At Dumfries, many of the objections above adverted to have been surmounted by the erection of a separate building for the paupers, adjacent to the original structure, which is now appropriated to patients belonging to the better ranks of society only.

"As regards the classification and separation of the patients, in reference more particularly to their mental condition, we are of opinion that the distinctions and subdivisions are too minute and special, and that the different classes of patients are not sufficiently associated together.

"At Dumfries, the demand for the admission of those in less affluent circumstances has been laudably met, by appropriating some of the rooms originally designed for the wealthy classes to the use of patients having only moderate resources. It appears, from the evidence of Dr. Browne, that two important results have followed the adoption of this plan—the first as respects the condition of the patients, and the other as respects the funds of the establishment. He states that not only are the patients paying moderate rates placed under more favourable circumstances as regards treatment and prospect of recovery, by being associated together, but, further, that the establishment derives more benefit from such a class, in a pecuniary point of view, than from patients who pay much larger sums for separate accommodation.

"The steps lately taken at Montrose, by the resident physician, in order to afford increased accommodation for patients of the middle class, by giving up a portion of his private residence for their use, are well worthy of commendation.

"In the chartered asylums generally, few or no limitations are made as regards the nature of the cases for which admission is requested; and patients are very seldom refused on account of epilepsy, pregnancy, the long duration of the malady, or the violent conduct of the patient. Occasionally, however, cases sent by Procurators-Fiscal, or removed from prisons, are rejected, on the ground that the effect upon the other patients would be prejudicial.

"Considerable facilities are afforded for the ready admission of patients, and no regulations are adopted for restricting their reception to particular days. Neither is it necessary to bring them in the first instance before the committee.

"As an instance of the confidence reposed in the medical superintendents of chartered asylums, and as an indication that some diminution has taken place in the repugnance to asylums, which hitherto has been, and still is so prevalent, we may mention that in many of the asylums, a number of individuals have voluntarily presented themselves for admission. They are generally cases of relapse, and frequently persons having a suicidal or destructive propensity, who, feeling certain premonitory symptoms, well known to themselves as the precursors of an attack of mania, at once take the judicious step of placing themselves under care and treatment."

In concluding this portion of their Report, the Commissioners judiciously observe that, at several public asylums, there appear some rather remarkable distinctions as respects the remuneration of officials. The disproportion between the sums paid to the secretary and treasurer of each institution is the most striking. At Edinburgh, Dumfries, and Glasgow—all large asylums—the matrons actually receive *higher salaries* than the assistant medical officers. This is wrong, if not unjust; and, in our estimation, such practices ought to be altered. Not only should every member of the medical staff be better remunerated than occurs now in several instances, but, to quote our correspondent, Dr. Webster, when adverting to these and similar matters,—

"The matron—who is sometimes too highly salaried, in relation to other officials and her actual position—appears frequently not sufficiently subordinate. This objection has been felt elsewhere; and in France, for example, where they manage many things often so well in lunatic asylums, a lady matron is almost unknown. Throughout Scotland, as also in England, sufficient attention is not invariably paid to their qualifications in the character of housekeepers, head attendants, and as sick nurses, when the governors select for appointment this occasionally rather too self-important personage."

These remarks deserve attention, and have been here reproduced for the special consideration of managing committees and public authorities who superintend Scottish chartered lunatic asylums.

According to particular extracts in our previous pages, the Commissioners seem, on the whole, to have been much satisfied with various public institutions for the insane in Scotland, as likewise regarding their general management. In reference, however, to licensed houses for receiving lunatics, they found great varieties in regard to accommodation and treatment, although the rates of payment were much the same in amount. These private establishments—many being chiefly for pauper patients —are situated principally in the neighbourhood of Edinburgh, viz., fourteen near Musselburgh, with several in Lanarkshire. The reason why so large a proportion of such licensed houses have been opened in the vicinity of the Scottish metropolis, is considered to be accounted for by the great facility with which permissions are obtained from the authorities of Mid-Lothian to receive and treat lunatics. These institutions vary considerably in size: some contain only one lunatic, and others nearly ninety. A few admit none but private patients, several only insane paupers; while the majority take both classes as inmates of their establishments.

Alluding generally to licensed houses for the insane in Scotland, the Report states:—

"The proprietors of some of the houses receiving patients belonging to the upper grades of society, are men of education, and well fitted, by professional training, to have the management of institutions for the insane. But, as a class, those who receive pauper patients are totally unfit for the proper discharge of the highly responsible and delicate duties they undertake.

"Licences have been conceded to persons who have no knowledge whatever of the nature or treatment of insanity, who have not even the experience of an ordinary nurse in a general hospital, and who are, besides, unprovided with sufficient capital to make a satisfactory provision for all the wants of those under their charge. Thus, at Musselburgh, we found one proprietor whose previous occupation had been that of a victual-dealer; another had been an unsuccessful baker; another had been a gardener; and the last person who had obtained the Sheriff's sanction for a licence, was a woman keeping a public-house, who had taken a second house for the reception of lunatics, with the view, as we were told by her daughter, of keeping both for a while, and continuing that which should prove the more successful speculation."

With reference to construction, and the accommodation supplied at various institutions, adverted to by the Commissioners, it appears,—

"With the exception of the convalescent department of Saughtonhall, none of the houses occupied as private asylums were originally built for the purpose. In one or two of the better class, such as Saughtonhall and Whitehouse, great expense has been incurred by the proprietors in providing suitable accommodation for the patients; but generally a private house has been rented, or bought, and afterwards altered and enlarged, to fit it (in most cases very imperfectly) for its new destination. The sole aim, especially in the houses where the patients are principally paupers, has evidently been to accommodate the greatest possible number at the smallest outlay. Hence outhouses, which were never intended for human habitations, have, in some cases, been filled with beds, and used as accommodation for patients both by day and night. In other asylums, such as that of Langdale, large dormitories have been built, sufficiently spacious for the number of occupants, but bare, comfortless, and insufficiently furnished. In other cases, again, every room is overcrowded, and houses of moderate size are made to accommodate a surprisingly large number of patients. Thus, the licensed house of Lilybank, at Musselburgh, which is rented at 35*l*. a year, has a population of 73 patients, besides the family of the proprietor, and the attendants. Frequently, also, there is no proper separation of male and female patients, who are placed in adjacent apartments approached by the same stair or passage, who use the same airing-courts, and are not even provided with separate water-closets.

"Another consequence of the prevailing tendency to economise, is a general want of furniture, and in several instances an almost total absence of everything that is not absolutely necessary, and even of articles that among the poorest people are considered indispensable. Most of the pauper houses have no day-rooms, the patients, when not in the airing-grounds, occupying their crowded sleeping-rooms during the day. These rooms are, for the most part, unprovided with seats, and the beds are used as substitutes. There are commonly no tables, and the meals are served in the most slovenly manner. The patients eat their food seated on the beds, or squatting on the floor of their rooms, or in corners of the airing-courts."

Upon the important subject of medical attendance at the private establishments now under review, the following remarks are made, but which, it is to be hoped, may be overdrawn, and apply only to exceptions:—

"In two or three instances, the proprietors of licensed houses are medical men, who conduct their establishments without any other medical aid; a practice which, under the present system of imperfect supervision, is open to objections, especially in the case of pauper houses, as it leaves the treatment of the patients entirely in the hands of parties whose pecuniary interests run counter to a liberal treatment of the patients. In other cases, the independence of the medical attendant is affected by his being liable to dismissal, should he place his opinion in antagonism to the views of the proprietor. He, in fact, holds the appointment only while it pleases the latter to retain his services, and hence his power of remedying abuses is greatly circumscribed. The existence of improper practices would, we conceive, be in some measure checked, if the medical attendant of a licensed house had greater authority, and if he were placed in a more independent position. At present he is appointed and dismissed at the pleasure of the proprietor. His duties are not defined; he does not regulate the diet or exercise—does not examine the clothing or bedding; in fact, he takes little or no part in directing the moral treatment of the patients, his functions being chiefly confined to prescribing in case of bodily illness. In some houses, two medical gentlemen are in the habit of attending, each taking charge of a certain number of patients; but generally the proprietor orders the shower-bath seclusion, or mechanical restraint, to be applied at his own discretion, without even consulting them. The consequence is, that mechanical coercion is applied and continued in these houses to a considerable and much greater extent than is known to the medical officer."

Notwithstanding the interesting discussions which have prevailed recently, and for many years, throughout England respecting physical coercion in cases of insanity, which have ended in its now almost total rejection, as not only cruel treatment, but most injurious to patients whenever employed, matters appear very different in Scotland, at some private receptacles, to what they are found to be in public or chartered institutions. For, when alluding to the use of mechanical means and seclusion, the Royal Report says—

"Instrumental restraint is in very general use in all the pauper houses, and not unfrequently also in the houses for private patients. There are houses in which some of the paupers are constantly manacled, either with the view to prevent their escape, or to keep them from attacking the attendants or patients. The strait-waistcoat is in daily use. The cause of this large amount of mechanical restraint appears to be chiefly due to the very small number of attendants, to deficient exercise, and to the great want of small rooms for the temporary separation of excited patients. Notwithstanding any regulations to the contrary, we have reason to think that, in most of the licensed houses, the attendants have the power of applying restraint at their discretion.

In almost every house, we found handcuffs, leg-locks, gloves, straps, and strait-waistcoats, and these not in the custody of the proprietor or medical attendant, but hanging up in the wards, or in the rooms of the attendants, who were evidently without any check as to their application, showing that the practice of restraint is still very prevalent. We may here mention the fact, that, in the early part of the present year, one of the principal cutlers in Edinburgh applied at the Morningside asylum for a pattern of the manacles and leg-locks used there, to enable him to execute an order he had received from one of the houses in Musselburgh. It is almost needless to remark that the superintendent was unable to comply with the request.

"Seclusion rooms are attached to some of the licensed houses. In one or two they are understood entirely to supplant physical restraint, but usually they are supplementary to it, and patients confined in them are sometimes also mechanically restrained. They are generally located in outhouses, and are frequently without the means of warming and ventilation."

Without entering any further, at present, into various collateral questions of interest connected with the establishments now under discussion, as the subject is by no means agreeable, the following summary, drawn up by the Commissioners, may be quoted to indicate the views they arrived at, or entertained, after due inquiry and personal inspection:—

"The bedding is of a coarse and cheap description, insufficient in quantity, and it is not renewed when filthy—whereby a saving of materials is effected. A further saving is also obtained by making one bed serve for two, and even three patients.

"The beds, in some cases, serve the purposes of seats; there is a general want of tables, and utensils necessary in a household, and of articles needed by the sick and infirm, as well as of books, and other means of amusement. Thus, in respect of furniture, &c., very little outlay has been made. By crowding the patients together day and night, the expense of fuel is diminished.

"The inmates during the winter months pass the greater part of each 24 hours in their beds, whereby candle-light is saved. In Langdale asylum the patients are not allowed candle-light at any season.

"By removing the body-linen at night, and by the long use of articles without washing, the ordinary expense in wear and tear is prevented.

"Judging from the diet served to the patients, the expenditure in food must be small; few extra articles of diet are provided, and little or no tobacco is allowed the patients.

"As respects service and wages, the employment of mechanical restraint, as a substitute for watchfulness; the mode of diminishing labour by placing two patients to sleep in the same bed; the plan of keeping the patients in the yards, and thus obviating the necessity of employing a paid servant to accompany them in their walks, or to induce them to enter on some occupation—are obvious means of reducing the expenditure in these respects.

"In bathing, and means of personal cleanliness, in washing of clothes and bedding, the outlay appears to be very small and inadequate.

"With the above facts before us, we cannot doubt that, in many instances, practices obviously wrong, and detrimental to the patients, have been adopted in licensed houses, because an increased profit would thereby be obtained by the proprietor; and hence it may be well here to enumerate, in contrast to the deficiencies of the licensed houses, a few of the advantages offered in the chartered asylums, where the only motives are the welfare and benefit of the patients."

Poorhouses and prisons next occupy the Commissioners' attention. Here, likewise, while some things are spoken of approvingly, many were considered to require amendment. The lunatic wards are generally small and ill-contrived, while economy is what the parochial authorities chiefly study in various instances. The accommodation afforded seemed often insufficient, the rooms being badly furnished, and little if any attention paid to giving a cheerful prospect to such dwellings. The usual medical attendant appears frequently too dependent on the parties appointing him to the office he fills, to be able to perform his professional duties satisfactorily. Although there sometimes prevails overcrowding, the clothing of inmates is generally of a good description. Personal cleanliness, they say, seems attended to tolerably; while the diet in several poorhouses appeared even superior to that of ordinary paupers. At some places the proportion of recoveries was high, but in these receptacles the mortality of patients was reported as usually greater than the chartered public asylums generally exhibited.

On the other hand, the treatment which lunatic inmates received in prisons was reported to have been generally as good as circumstances allowed; although occasionally it is considered that some lunatic prisoners have been roughly treated, of which an example is mentioned.

The condition of criminal lunatics is afterwards noticed. The total number of this class is not large, and all are located in the general prison at Perth, with one exception—who, from some unascertained cause, has been left in the Edinburgh Asylum. Those patients now in the Perth gaol amount to 28, of whom 22 are males and 6 females. Respecting this category, or insane criminals, so enumerated, the Report says—

"In the treatment of the criminal lunatics at Perth, a sufficient distinction is not made between disease and crime. An individual who, in a state of insanity, commits an offence, and who, by the verdict of the jury, is acquitted of the guilt of crime, is, nevertheless, there treated as a criminal, in so far, that he is kept in confinement, not as a patient, but as a prisoner; and is, in accordance with this distinction, deprived of those means of treatment which might have conduced to his recovery—or, if this were impossible, have alleviated his condition.

"In the lunatic wards of the prison, there are no proper means of classification; and all the patients, of whatever condition in life, must associate together. There are two airing-courts for the males, and one for the females, all of very moderate size; but, apart from them, there is an almost total want of the means of occupation and amusement. There may be sound reason in not allowing a criminal prisoner to better his condition by the expenditure of his private means; but it seems unnecessarily harsh to place an insane patient in circumstances where he is deprived of comforts with which his friends are willing to provide him, and which would be supplied in an ordinary asylum. Viewed in this light, the placing of criminal lunatics, whose means are sufficient to pay the rates of the chartered asylums, in the cells of the lunatic wards of the Perth prison, is virtually punishing the lunatic as a criminal, for acts for which he has either not been tried, or for which the jury has declared him not responsible."

Besides the lunatic convicts just mentioned, it is further stated that

"To this number ought to be added, as belonging to the same category, those lunatic convicts who are sent back to local prisons for the purpose of being liberated at the expiry of their sentence, and who, on liberation, are again immediately arrested and conveyed to asylums. What the number of these may amount to, we have no means of forming an accurate estimate, for the prison authorities on their liberation make no inquiry as to their future disposal, nor do the asylums to which they are conveyed make any investigation into their past history. There is here a complete break, which arrests all further attempts at accurate research. It is, indeed, one of the greatest defects of the present system of treating lunatic convicts, that, at the expiry of their term of imprisonment, they are discharged without any proper provision being made for their future care."

Suggestions as to the treatment and disposal of criminal lunatics are subsequently discussed, into which we do not now enter. In recapitulation of this subject, moreover, the Commissioners subsequently observe that

"The number of such lunatics in Scotland is too small to require the erection of a special asylum for their reception; while, on the other hand, it is too great to warrant the continuance of the present system, which fulfils no purpose beyond that of security. It must, however, be borne in mind that the future number of criminal lunatics will greatly depend upon the manner in which it shall be determined hereafter to dispose of convicts—whether they shall be retained in prisons in this country, or be transported beyond seas. The small portion of female criminal lunatics in the wards of the General Prison at Perth is, no doubt, partly owing to the small number of female convicts hitherto retained in Scotland."

The condition of insane patients not resident within asylums is next investigated in the Report; the first being pauper lunatics living with relatives or strangers. Private insane patients residing with relations, or those in no way connected with the lunatics, are adverted to subsequently. This insane class is numerous; only ten living in houses reported to the Sheriff, while not less than 1800 such unfortunate cases are in no way officially recognised. As illustrations of the improper proceedings which seem to prevail, without any licence or warrant from public authority, in too many examples, the Report says that, in general, only one patient occupies the same house, but occasionally it is otherwise. For instance—

"One of these houses is situated at Trinity, near Edinburgh. It is intended, principally, for the reception of ladies addicted to intemperance; but they are detained against their will, and measures are, accordingly, taken to prevent their escape. The windows of the house are all barred, the front gate is kept constantly locked, and the ladies are very seldom allowed to go beyond the court and garden, which contain only a few square yards of ground. The house was visited on 12th July, 1855, and then contained three patients,—one of them a male, suffering from general paralysis. The second house is situated in the village of Laurencekirk in Kincardineshire, and contained, when visited on October 1, 1855, two insane and two sane boarders—all females. This house, we were informed by the proprietor, is known to the Sheriff of the county as one receiving lunatics; but he does not consider it necessary to grant licences, or to visit it. Another house was mentioned to us as existing at Haddington; but we found, on visiting that town, that it had been closed for some time—it was said in consequence of the death of the wife of the proprietor. Although the above are the only private unlicensed houses to which our attention has been specially called, we have reason to think that others exist in Skye, and similar remote districts."

When further illustrating this part of their inquiry, the Commissioners state—

"Unlicensed houses have been opened, as trading concerns, for the reception of certain classes of patients, who are detained in them without any safeguard whatever against ill-treatment or abuse.

"A very large number of single patients are detained at home, or illegally placed in the houses of strangers. The generality of these are in a most destitute condition, being badly lodged, ill-fed, scantily clothed, and not provided with sufficient bedding. A few are subjected to personal chastisement, some are permanently chained, others are placed in outhouses, or are locked up in small closets just capable of holding them. Many are filthy in their persons, infested with vermin, covered by mere rags, or allowed to remain perfectly naked. Some are without bedding, except loose straw or heather cast on rough boards, and their rooms emit an intolerable stench. Others, again, are homeless, and are allowed to wander at large.

"A considerable proportion of the weak-minded females have borne illegitimate children, and in many of these instances the mental imbecility is apparent in the progeny. Not only are many of the single patients grossly neglected, but many of them are a great charge and source of anxiety to their relatives, and a cause of apprehension to the public. In remote districts, the patients are generally allowed to remain without appropriate treatment till the malady has become incurable, and only when troublesome or unmanageable are they sent to an asylum, always at a great distance from their homes and relatives. They are often harshly treated, and during the journey to the asylums are frequently painfully manacled, or secured with ropes, sometimes bound so tightly as to penetrate the flesh; and cruelties of this kind appear to pass unnoticed and unpunished. They are recklessly transported from one place to another, and sometimes brought from remote districts, and shamefully cast free among the population of large towns, to get rid of the expense of their maintenance."

But our analysis of the very important official document, now brought under the notice of readers, must here draw to a close. Other interesting and important matters of inquiry might doubtless be made the subject of additional instructive discussion, had not the limited space presently at command been already exhausted. Therefore, although many other valuable extracts could easily be here made from the official report in question, and which truly merit careful perusal, we refrain, while seriously recommending those parties in authority throughout Scotland, who take an interest in such matters, or have been referred to in the document itself, seriously to read the observations there made on various subjects investigated. Finally, we would now conclude by directing attention to the specific remedies and legislative measures suggested in the following summary, wherewith the Scotch Commissioners terminate their Report:—

"1. The erection of district or county asylums for pauper lunatics, including accommodation for the insane belonging to the labouring classes, who are not strictly paupers. Likewise, more suitable accommodation for criminal lunatics.

"2. Means for ensuring greater caution and discrimination as regards the licensing of houses for the reception of the insane; for imposing some check upon the licensing of new houses; and for conferring powers to close those already opened for paupers, so soon as public asylums shall have been erected; or, at any other time, if not properly conducted.

"3. Regulations, by which all pauper lunatics, not in asylums, shall be brought under proper visitation and care, and periodical reports be made, as to their condition, by medical men, so as to afford a safeguard against abuse and ill-treatment, and secure the ready and careful transmission of all proper cases to asylums.

"4. An accurate definition of the powers and duties of Sheriffs, in reference to the insane, so as to secure a more uniform practice and united action amongst them.

"5. Rules for the guidance of the Board of Supervision, parochial boards, inspectors of poor, and district medical officers, in all matters relating to the management of the insane.

"6. More complete regulations, in reference to medical certificates; to prevent interested parties signing them; to specify the length of time the document shall remain in force; and to require a statement of the facts or evidence upon which the opinion as to the patient's insanity is founded. Also a limitation of the time during which the Sheriff's order shall remain in force, previous to the admission of the patient, and also in case of escape.

"7. The formation of a complete system of schedules and returns, together with full records of all admissions, discharges, deaths, and accidents. Also the institution of registers and case books, showing the medical treatment pursued in each case, and whether, and to what extent, restraint and seclusion were employed.

"8. Comprehensive regulations applicable to licensed houses and poorhouses, while continuing to receive lunatics, for securing to the patients sufficient medical and other attendance; kind and appropriate treatment; proper diet, clothing, bedding, exercise, and recreation; and adequate means of religious consolation.

"9. A requirement that, on recovery, patients shall be discharged by the medical attendant of the establishment.

"10. Restrictions on the removal of pauper patients by inspectors before recovery.

"11. Precautions for preventing injustice in transporting aliens.

"12. Better regulations as to dangerous and criminal patients.

"13. Measures by which persons labouring under insanity may voluntarily place themselves under care in an asylum.

"14. Special regulations for prolonging control over cases of insanity arising from intoxication.

"15. Enactments for extending further protection to the property of lunatics, and for ensuring the proper application of their funds.

"16. The imposition of suitable penalties for infringement of the law, and power to modify them according to circumstances.

"17. Powers to raise sufficient funds for the purposes of the Act.

"18. The creation of a competent Board, invested with due authority, and to whom the general superintendence of the insane in Scotland shall be intrusted; including powers to license houses for the reception of the insane; to visit all asylums, licensed houses, poorhouses, and houses containing only single patients; to order the removal of patients to or from an asylum, or from one asylum to another; to give leave of absence to convalescent patients; to regulate the diet in asylums and licensed houses for pauper patients; to make regulations for their management, &c., &c.; with direction to report to the Secretary of State for the Home Department.

"19. The formation of local boards for the management of individual asylums, who shall act in conjunction with the General Board."

The large appendix accompanying the Blue Book, which has now been the subject of our critical remarks, is really a voluminous production. It contains, spread over 581 pages, much very interesting historical matter, regarding the origin, date of opening, subsequent progress, and recent management of the various chartered asylums of North Britain, besides numerous statistical details, illustrating each establishment. Farther, it also gives the evidence of different parties therewith connected, and belonging to or interested in private receptacles for the insane who were examined; and whereupon the Commissioners, of course, have virtually based their subsequent recommendations.

After the facts thus officially published have been maturely weighed, but without any leaning either in favour of, or against the parties chiefly implicated, impartial persons will then be fully able to form an unbiassed opinion in reference to the justice or necessity of the different propositions now authoritatively recommended.

